# Do Dietary Habits Influence Trace Elements Release from Fixed Orthodontic Appliances?

**DOI:** 10.1007/s12011-017-1011-5

**Published:** 2017-04-11

**Authors:** Paulina Wołowiec, Katarzyna Chojnacka, Bartłomiej W. Loster, Marcin Mikulewicz

**Affiliations:** 1 Department of Advanced Material Technologies, Faculty of Chemistry, Wroclaw University of Technology, ul. Smoluchowskiego 25, 50-372 Wrocław, Poland; 2 Department of Orthodontics, Dental Institute, Faculty of Medicine, Medical College, Jagiellonian University, Cracow, ul. Montelupich 4/108, 30-383 Kraków, Poland; 3 Department of Dentofacial Orthopaedics and Orthodontics, Division of Facial Abnormalities, Medical University of Wrocław, ul. Krakowska 26, 50-425 Wrocław, Poland

**Keywords:** Orthodontic appliance, Chromium, Nickel, Dietary habits, Hair mineral analysis

## Abstract

The objective was to investigate the effect of dietary habits on the release of Cr and Ni ions from orthodontic appliances by hair mineral analysis. Patients (*N* = 47) underwent electronic questionnaire survey to investigate the effect of dietary habits on Cr and Ni levels in hair. The research was carried out on hair sampled at the beginning and in the 4th, 8th, and 12th months of the treatment. The content of Cr and Ni in the collected samples was determined by ICP-OES. The study showed that consumption of acidic dietary products may have the effect on increasing the release of Cr and Ni ions from orthodontic appliances. The release of Cr from orthodontic appliances in patients who consumed fruit juice, coffee, yoghurt, and vinegar was higher. The coefficients enabling comparison of metal ions release pattern at a given sampling points were defined. The comparison of the coefficients yielded the information on the possible magnification of metal ions released as the result of the additional factor consumption of acidic food or drink that intensifies metal ions release. The following magnification pattern was found for chromium: coffee (7.57 times) > yoghurt (2.53) > juice (1.86) > vinegar (1.08), and for nickel: vinegar (2.2) > coffee (1.22) > juice (1.05). Yoghurt did not intensify the release of nickel. Concluding, orthodontic patients should avoid drinking/eating coffee, yoghurt, fruit juices, and vinegar.

## Introduction

Dietary habits affect well-being and health [1]. The increased consumption of soft drinks has raised several concerns about the health consequences such as obesity, diabetes, hypocalcemia, dental caries, dental erosion, and mental health problems [2, 3].

Many papers investigated the effects of acidic beverages on dental erosion [4–8]. This is the result of the enamel and dentin susceptibility to exogenous acids, originating from acidic food (fruit, yoghurt) or beverages (fruit juice, energy drinks, cola drinks) [4, 9]. Soft drinks contain several acids such as phosphoric, citric, tartaric, lactic, and maleic acid [10]. Their pH may be close to 2.0 or 3.0, for example, Coca-Cola® 2.3, Pepsi® 2.3, Sprite® 2.7, Red Bull® 3.1, Powerade® 3.1, orange juice 3.7, white wine 3.0 [4, 5, 7].

Acidic foods and drinks not only have influence on tooth enamel but also reduce life time of dental restorations [9]. It has been reported that soft drinks affect the decrease of mechanical properties of restorative materials, especially surface hardness, surface integrity, and solubility [11–13]. Similarly, energy drinks influence the surface hardness of these materials [14]. However, damage was not correlated to pH values of beverages, but it was associated with their chemical composition [11, 13]. Apple and orange juice were more deteriorating than Coca-Cola® [11, 13]. Additionally, it was reported that drinks (e.g., coffee, tea, red wine, Coca-Cola®) and colored foods (e.g., ketchup, mustard, soy sauce) may cause color change of restorative materials [15, 16].

Dietary habits may adversely affect orthodontic treatment by reduction in the shear bond strength of brackets, increased risk of dental caries and enamel microhardness, and change of color stability of orthodontic adhesives and elastic ligatures [3, 17–20]. In the current literature, the number of the studies related with the impact of acidic foods and soft drinks on the corrosion of orthodontic appliances and release of metal ions is very limited. Hair mineral analysis can be a useful tool in the determination of metal ions release from orthodontic alloys [21].

The aim of the present study was to investigate the effect of dietary habits on the release of Cr and Ni ions from orthodontic appliances by hair mineral analysis. The approach towards simplified modeling metal ion release in response to various dietary factors was applied. The goal was to elaborate dietary recommendations for orthodontic patients, in order to diminish the negative effects related with intensified metal ions release by dietary factors. Although many papers on metal ions release from orthodontic appliances in in vivo systems have been published [22, 23], including evaluation of cytotoxicity and genotoxicity in orthodontic patients [24–26], no studies taking into account the organism of a patient as a whole have been conducted. This justifies the use of hair mineral analysis.

## Materials and Methods

The study was performed in accordance with the principles laid down in the Helsinki Declaration. The study was carried out with the approval of the Ethical Committee of Wroclaw Medical University (KB-400/2010). Hair was sampled from 47 patients (16 males and 31 females), average age 17.2 ± 6.6 years. The population consisted of individuals who underwent orthodontic treatment with fixed appliances and filled out electronic questionnaire, which concerned the individual characteristic (e.g., general information, dietary habits, environmental exposure). The hair samples were collected at the beginning of the treatment and after 4, 8, and 12 months. The details of the experiment were described earlier [21].

### Analytical Methods

A sample (0.5 g) was purified from organic matter with concentrated nitric acid—69% m/m (5 mL), spectrally pure (Suprapur, Merck, Darmstadt, Germany) in Teflon bombs in microwave oven Milestone Start D (Sorisole, Italy). The selected parameters of the process assured the complete digestion of samples. After mineralization, samples were diluted with double-demineralized water (Millipore Simplicity, Molsheim, France) to 50 g. The concentration of chromium and nickel was determined by ICP-OES Varian-Vista MPX (Australia), equipped with ultrasonic nebulizer CETAC U5000AT+. The analyses were carried out in quality management system according to PN-EN ISO/IEC 17025:2005 (accreditation number AB 696 PL PCA). Quality assurance of the test results was achieved by using certified reference material—Human Hair NCS ZC81002 from the China National Analysis Centre. The samples were analyzed in three repeats (the reported results of analyses were arithmetic mean, and the relative standard deviation was <5%).

### Statistical Methods

The results were elaborated statistically by *Statistica ver. 10.0*. Descriptive statistics (means, standard deviations) were reported. Normality of distribution of experimental results was assessed by Shapiro–Wilk test. Statistical tests were performed by the analysis of variance with repeated measurements using the Bonferroni test. Results were considered significantly different when *p* < 0.05.

## Results

The effect of dietary habits on the release of Cr and Ni ions from orthodontic appliances was investigated basing on the results of electronic questionnaire survey and hair mineral analysis. The differences between the level of elements in hair sampled during the treatment and the level of metals in hair sampled at the beginning of the treatment were assessed by the Bonferroni test. It was found that after insertion of orthodontic appliances, the Cr and Ni contents increased in hair of patients. Statistically significant differences were found for Cr in hair of patients, who did not consume vinegar (Table 1). The mean concentrations were eight to nine times higher during the treatment than before the beginning of therapy.

**Table 1:** The effect of dietary habits on contents of chromium in hair collected before and during orthodontic treatment (mg/kg)

Dietary habit		Duration of orthodontic treatment (month)											
		0			4th			8th			12th		
		Mean	SD	*N*	Mean	SD	*N*	Mean	SD	*N*	Mean	SD	*N*
Juice	No	0.0266	0.0521	24	0.192	0.369	24	0.178	0.328	22	0.168	0.314	19
	Yes	0.0134	0.0306	23	0.176	0.281	23	0.179	0.258	23	0.150	0.266	20
Yoghurt	No	0.0212	0.0426	9	0.0620	0.0943	9	0.0862	0.145	9	0.124	0.165	8
	Yes	0.0199	0.0437	38	0.213	0.354	38	0.202	0.315	36	0.167	0.312	31
Coffee	No	0.0276	0.0511	31	0.158	0.267	31	0.151	0.263	29	0.140	0.239	25
	Yes	0.00578	0.0110	16	0.235	0.422	16	0.288	0.340	16	0.191	0.365	14
Vinegar	No	0.0173*^,^ **^,^ ***	0.0389	39	0.131**	0.237	39	0.154***	0.248	37	0.158*	0.258	32
	Yes	0.0338	0.0608	8	0.446	0.544	8	0.292	0.445	8	0.161	0.422	7

Bonferroni test: *(*p* ≤ 0.05); **^,^ ***(*p* ≤ 0.1)

**Fig. 1:**
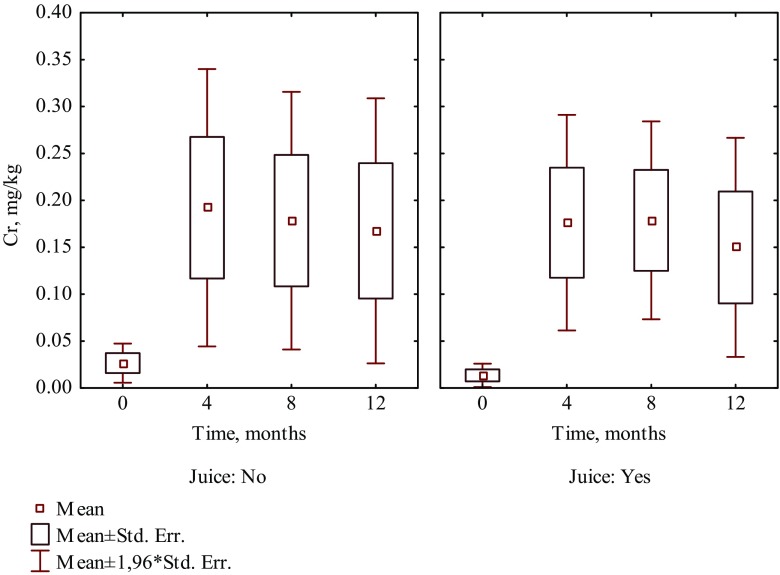
The effect of drinking juice on the content of Cr in hair of patients in time during 1 year of orthodontic treatment

**Fig. 2:**
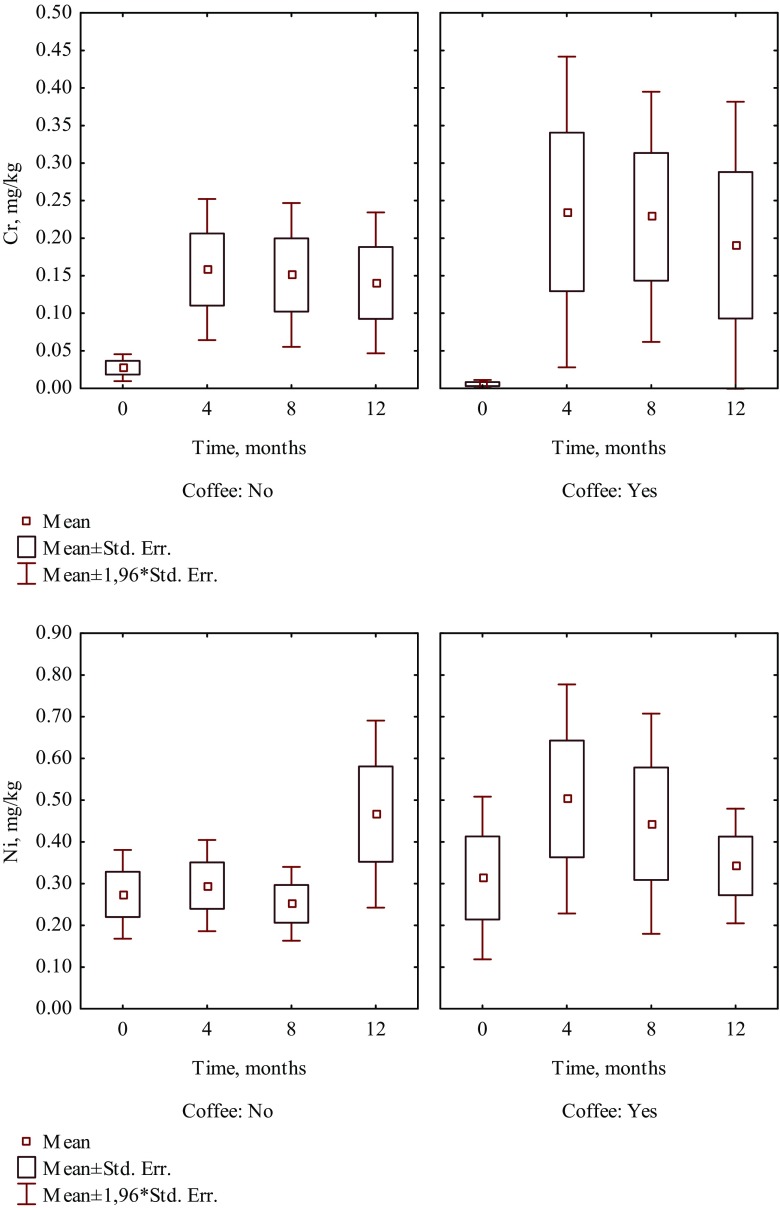
The effect of drinking coffee on the content of Cr and Ni in hair of patients in time during 1 year of orthodontic treatment

**Fig. 3:**
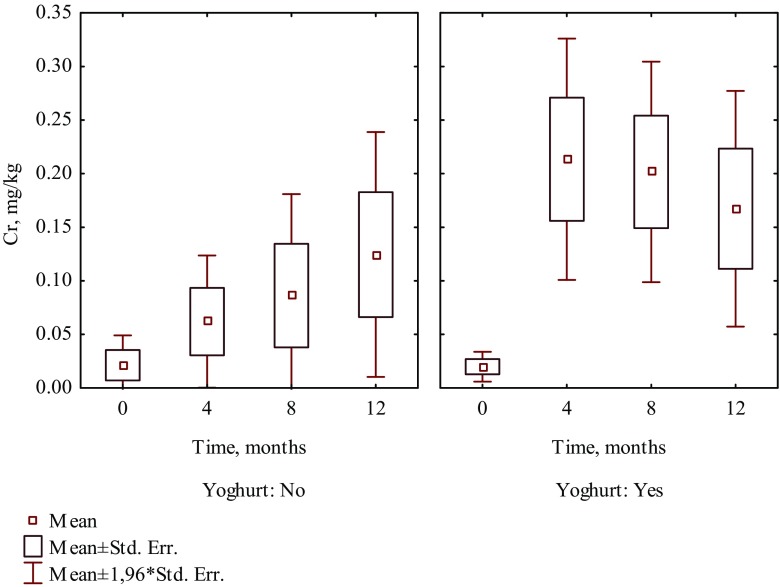
The effect of consuming yoghurt on the content of Cr in hair of patients in time during 1 year of orthodontic treatment

**Fig. 4:**
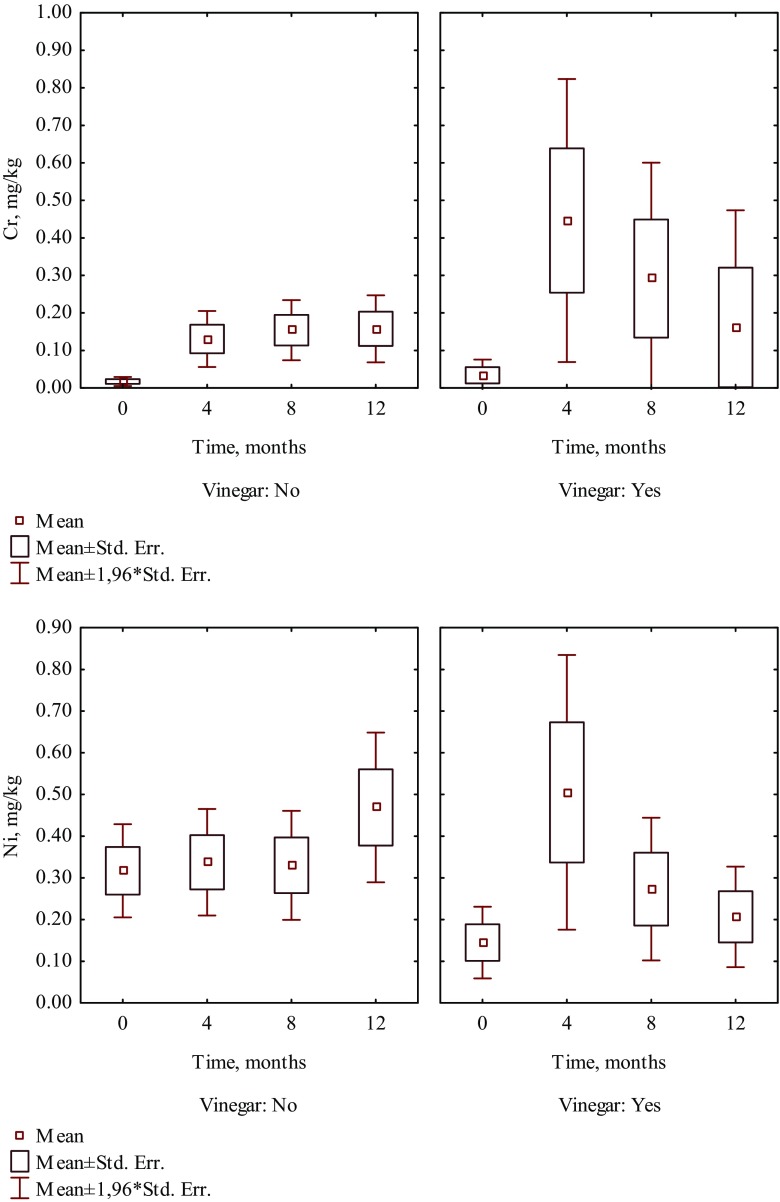
The effect of consuming vinegar on the content of Cr and Ni in hair of patients in time during 1 year of orthodontic treatment

It is worth noting that in patients who consumed the selected food products such as fruit juice, coffee, yoghurt, and vinegar, a higher increase of Cr and Ni levels was observed than in hair of patients who did not consume those products (Tables 1 and 2). Four months after beginning of treatment, the content of Cr increased 13 times in hair of patients who were drinking juice, whereas in patients who did not drink juice, it increased seven times (Fig. 1). The intake of coffee caused the increase of Cr and Ni levels in hair almost 41 times and 60.2%, respectively, while in hair of patients who did not drink coffee increased nearly 6 times and 7.6%, respectively (Fig. 2). The content of Cr increased almost 11 times in hair of patients who included yoghurt in their diet, whereas in patients who did not eat yoghurt, it increased nearly three times (Fig. 3). The intake of vinegar contributed to the increase of Cr and Ni levels in hair more than 13 times and more than three times, respectively, while in hair of patients who did not use vinegar, they increased almost seven times and 6.3%, respectively (Fig. 4). Also, multiple linear regression analysis was used to identify the factors associated with content of Cr and Ni in hair of patients in time during orthodontic treatment. The results showed that the level of Cr was dependent on the treatment time, while the Ni content was not associated with both treatment time and dietary habits.

**Table 2:** The effect of dietary habits on contents of nickel in hair collected before and during orthodontic treatment (mg/kg)

Dietary habit		Duration of orthodontic treatment (month)											
		0			4th			8th			12th		
		Mean	SD	*N*	Mean	SD	*N*	Mean	SD	*N*	Mean	SD	*N*
Juice	No	0.331	0.396	24	0.443	0.449	24	0.323	0.303	22	0.500	0.639	19
	Yes	0.243	0.254	23	0.286	0.377	23	0.316	0.450	23	0.348	0.261	20
Yoghurt	No	0.131	0.056	9	0.222	0.238	9	0.273	0.256	9	0.354	0.312	8
	Yes	0.324	0.361	38	0.400	0.446	38	0.332	0.408	36	0.439	0.521	31
Coffee	No	0.274	0.302	31	0.295	0.310	31	0.252	0.243	29	0.466	0.571	25
	Yes	0.314	0.398	16	0.503	0.560	16	0.444	0.538	16	0.342	0.263	14
Vinegar	No	0.317	0.356	39	0.337	0.407	39	0.330	0.406	37	0.469	0.518	32
	Yes	0.145	0.124	8	0.505	0.475	8	0.273	0.247	8	0.207	0.163	7

Bonferroni test: *(*p* ≤ 0.05); **^,^ ***(*p* ≤ 0.1)

There were two distinctive patterns of metal ions release from orthodontic appliances in time as the results of eating/drinking acidic foodstuffs: (1) the level increased after insertion of the appliance and remained constant during the treatment or (2) decreased gradually, probably to the possibility of the formation of the passivation layer that would limit further solubilization of metal ions. The first group (no time effect) was observed for Cr and juice and for Cr and coffee. The second pattern (possible passivation layer) was observed for Ni and coffee, Cr and yoghurt, Cr and vinegar, and Ni and vinegar.

For the sake of interpretation of the results, the following coefficients were defined:

Release factor (RF) at a given sampling point that relates the content of metal (Cr or Ni) during specific time of orthodontic treatment to sampling before insertion of the appliance:

$$ {\mathrm{RF}}_{\mathrm{time}}=\frac{C_{\mathrm{time}}}{C_0} $$

This is the measure of the quantity of metal ions released during treatment.

The same factor was defined for the situation where the intake of specific food or drink occurred that could intensify the release of metal ions from the alloy of which orthodontic appliance was made:

$$ {\mathrm{RF}}_{\mathrm{time}}^{\mathrm{food}}=\frac{C_{\mathrm{time}}^{\mathrm{food}}}{C_0^{\mathrm{food}}} $$

Before the beginning of orthodontic treatment, there was a certain, background level of each element in hair of patients. During the study, a change in a level of a given metal in the organism, in relation to the consumption of specific food/drink, was investigated.

The comparison of those two coefficients provides the information on whether magnification of metal ions released occurred as the consequence of the presence of metal alloy in the oral cavity and an additional factor (acidic food or drink that could intensify metal ion release process). Therefore, if ${\mathrm{RF}}_{\mathrm{time}}^{\mathrm{food}}>{\mathrm{RF}}_{\mathrm{time}}^{\mathrm{food}}$, a given food or drink contributes to more intense metal ions release. This intensification was reflected as another magnification factor (MF):

$$ {\mathrm{MF}}_{\mathrm{time}}^{\mathrm{food}}=\frac{{\mathrm{RF}}_{\mathrm{time}}^{\mathrm{food}}}{{\mathrm{RF}}_{\mathrm{time}}} $$

${\mathrm{MF}}_{\mathrm{time}}^{\mathrm{food}}$ >1 signifies that the consumption of a given food/drink should be reduced during orthodontic treatment.

**Fig. 5:**
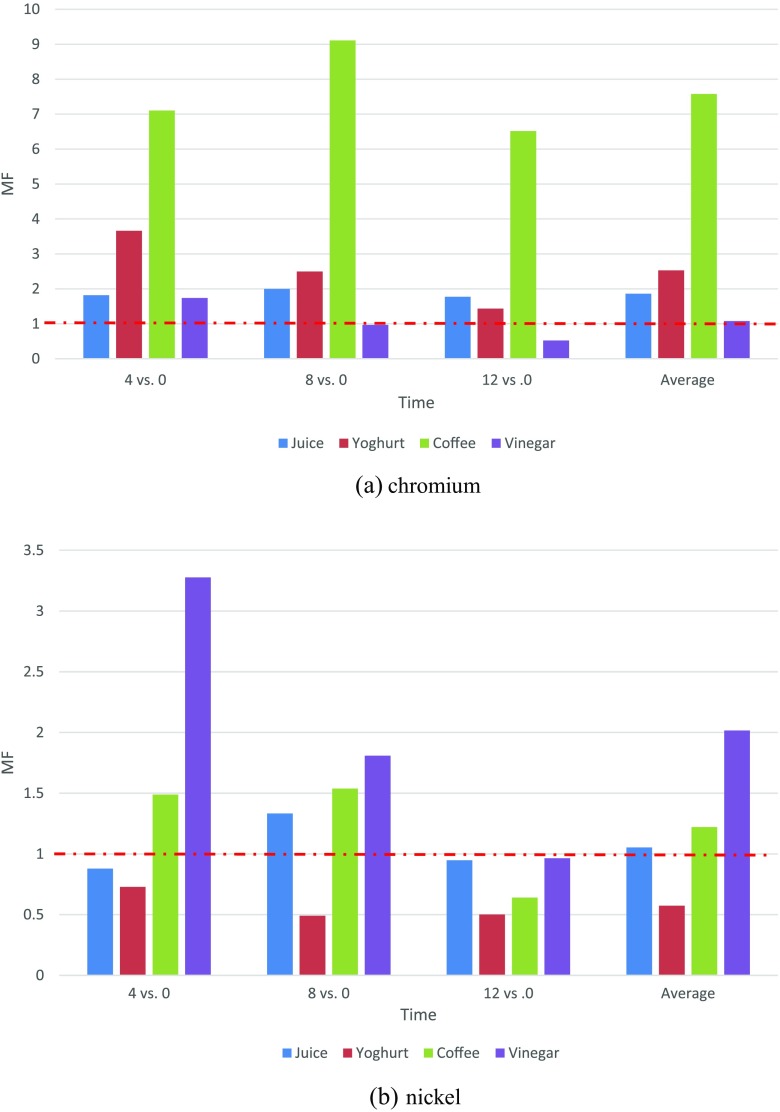
Magnification factor for foods/drinks. **a** Chromium. **b** Nickel

Figure 5 presents the values of RF and MF at a given sampling points for given foods or drinks and a given metal ion. Threshold line marks the value MF = 1. It was found that the magnification pattern for chromium was as follows: coffee (7.57 times) > yoghurt (2.53) > juice (1.86) > vinegar (1.08), and for nickel: vinegar (2.2) > coffee (1.22) > juice (1.05). Yoghurt did not intensify the release of nickel. This means that orthodontic patients should avoid drinking/eating coffee, yoghurt, fruit juices, and vinegar.

## Discussion

In the previous study, Mikulewicz et al. [21] reported that the content of Cr and Ni in hair of orthodontic patients substantially increased during the treatment. For Cr, the differences were statistically significant. The increase in Cr level was over nine times during the first months, while the increase in Ni content was not as strong, 27% after 4 months and 46% after 1 year. In the same study, effects of individual characteristics (e.g., gender, age) and dietary factors on the levels of Cr and Ni in hair collected before treatment were analyzed [21]. Other study suggested the possible increase of Ni and Cr in hair during orthodontic therapy [27].

In the present work, the results showed that increase in the content of Ni and Cr in hair during treatment was also dependent on patient’s diet. The food products such as fruit juice, coffee, yoghurt, and vinegar were characterized by low values of pH. According to literature, values of pH of those products were as follows: 2.1–3.6 fruit juice [7], coffee 5.0–5.5 [28], 3.8–4.6 yoghurt [29], and 2.5–3.5 vinegar [30]. Consumption of acidic food products can intensify aggressiveness of conditions of the oral cavity and can be the consequence of solubilization and increase the release of metal ions from orthodontic appliances.

Until now in the available literature, only a few papers concerning the influence of acidic environment on corrosion of orthodontic appliances have been published. Staffolani et al. [31] investigated the release of metal ions from orthodontic appliances in inorganic (pH 3.5–6.5) and organic acid solutions (*w*/*v* 1% each of tartaric, citric, and ascorbic acid at pH 2.2 or 1.5% each of lactic and acetic acid at pH 2.5). The authors reported that the release of Cr and Ni ions was few times higher in hydrochloric acid of pH 3.5 than in solution at pH 6.5. It was also shown that after the first day of incubation, Ni concentration was higher in organic acid solutions (1.9–2.5-fold) than in the hydrochloric acid of pH 3.5. In another study, Kuhta et al. [32] demonstrated that the concentration of Cr and Ni released from orthodontic appliances were on average 37 times higher in artificial saliva of pH 3.5 as compared to the artificial saliva of pH 6.75.

Schiff et al. [33] analyzed the influence of fluoride in certain mouthwashes on the risk of corrosion of wire-bracket pairs. The quantity of released Ni ions in mouthwashes was considerably higher than in artificial saliva (up to 100 times higher for the NiTi–CoCr pair). Also, an increase in Cr and Fe levels was observed, but it was not as strong as in the case of Ni [33].

In the study of Abalos et al. [34], corrosive action of soft drinks with low pH on the surface of Ni–Ti wires was reported. The effect was dependent on the surface pattern. Sajadi et al. [35] investigated the effects of Coca-Cola® and a non-alcoholic beer on the shear bond of orthodontic brackets and observed that there was a significant difference in the shear bond strength only for Coca-Cola®. Effects of acidic soft drinks on the shear bond of orthodontic brackets (Coca-Cola® and Sprite®) were evaluated also in an in vivo study. It was found that the Coca-Cola® group without resin infiltration showed the lowest resistance to shearing forces [36].

Kumar et al. [37] evaluated the release of nickel and chromium ions in human saliva during fixed orthodontic therapy, pointed out on more intense release during the initial phase, by measuring the level of metal ions in saliva (by ICP-OES). The levels of nickel and chromium were statistically significantly higher, while nickel showed a gradual increase in the first 10 days with a further decline. Chromium showed a gradual increase and was statistically significant on the 30th day of the treatment. Also, several other studies showed that the major release occurred during the first month of the treatment with further passivation [38]. This confirms the results obtained in the present study.

More detailed examination of the impact of dietary habits on the release of metal ions from orthodontic appliances can enable the development of nutritional recommendations for patients. These recommendations will reduce exposure of patients to Cr and Ni released from orthodontic appliances.

## Conclusions

The results suggest that consumption of food products of low pH (such as fruit juices, coffee, yoghurt, and vinegar) can intensify aggressiveness of conditions in the oral cavity and may have an effect on increasing the release of Cr and Ni ions from orthodontic appliances. Therefore, it would be useful to recommend to orthodontic patients to limit consumption of foods and drinks which are characterized by low values of pH to reduce the quantity of ions solubilized from metal alloys.
